# Intermolecular Structure Conversion-Based G4-TDF Nanostructures Functionalized μPADs for Fluorescent Determination of Potassium Ion in Serum

**DOI:** 10.3390/bios15040223

**Published:** 2025-03-31

**Authors:** Mengqi Wang, Xiuli Fu, Yixuan Liu, Zhiyang Zhang, Chenyu Jiang, Dean Song

**Affiliations:** 1 School of Chemistry and Chemical Engineering, Yantai University, Yantai 264005, China; 202200369065@s.ytu.edu.cn (M.W.); fuxiuli@ytu.edu.cn (X.F.); liuyx2025@foxmail.com (Y.L.); 2 Coastal Zone Ecological Environment Monitoring Technology and Equipment Shan-dong Engineering Research Center, Shandong Key Laboratory of Coastal Environ-mental Processes, CAS Key Laboratory of Coastal Environmental Processes and Ecological Remediation, Yantai Institute of Coastal Zone Research, Chinese Academy of Sciences, Yantai 264003, China; zyzhang@yic.ac.cn; 3 Suzhou Institute of Biomedical Engineering and Technology, Chinese Academy of Sciences, Suzhou 215163, China; 4 Tobacco Research Institute, Chinese Academy of Agricultural Sciences, Qingdao 266101, China

**Keywords:** G-quadruplex, DNA tetrahedral nanostructure, structure conversion, thioflavin T, potassium ion

## Abstract

Herein, we proposed a versatile G-quadruplex (G4)-tetrahedral DNA framework (G4-TDF) nanostructure functionalized origami microfluidic paper-based device (μPADs) for fluorescence detection of K^+^ by lighting up thioflavin T (ThT). In this work, TDF provided robust structural support for G-rich sequence in well-defined orientation and spacing to ensure high recognition efficiency, enabling sensitive fluorescence sensing on origami μPAD. After introducing ThT, the G-rich sequences extended from TDF vertices formed a parallel G4 structure, showing weak fluorescence signal output. Upon the presence of target K^+^, this parallel G4 structure transitioned to antiparallel G4 structure, leading to a significantly increase in fluorescence signal of ThT. Benefiting from the outstanding fluorescence enhancement characteristic of the G4 structure for ThT and excellent specificity of the G4 structure to K^+^ plus satisfactory recognition efficiency with the aid of TDF, this origami paper-based fluorescence sensing strategy exhibited an impressive detection limit as low as 0.2 mM with a wide range of 0.5–5.5 mM. This innovative G4-TDF fluorescence sensing was applied for the first time on μPAD, providing a simple, effective, and rapid method for K^+^ detection in human serum with significant potential for clinical diagnostics.

## 1. Introduction

Potassium ion (K^+^), as one of the indispensable species in the human body, plays a vital role in regulating numerous physiological processes, including nerve transmission, regulation of pH and blood pressure, enzyme activation, and maintenance of muscle strength [1,2,3]. The normal level of human serum K^+^ is around 3.5–5.0 mM [4]. Abnormal potassium ion concentrations may cause a variety of diseases, such as heart disease, high blood pressure, diabetes, kidney disease, stroke, and Addison’s disease [5,6]. Therefore, rapid and convenient sensitive detection of K^+^ is of significant importance. Toward this goal, many analytical methods have been established for the determination of K^+^, including inductively coupled plasma–mass spectrometry [7,8], atomic absorption spectroscopy [9,10], flame atomic absorption spectrometry [11,12], surface plasmon resonance [13,14], and ion chromatography [15,16]. Although these techniques are effective in detecting potassium ions, there are still obstacles that need to be overcome, such as their complex operation, time-consuming nature, and use of expensive instruments. Thus, it is necessary and meaningful to develop a simple and rapid method for the determination of K^+^.

To date, microfluidic paper-based analytical devices (μPADs) have attracted enormous interest owing to their outstanding detection advantages, such as high integration ability for performing “mixing–separation–washing–detection”, good biocompatibility, reduced sample and reagent consumption, low cost, higher portability, and simpler operation [17,18]. Moreover, by a chip pattern design, a variety of chip channels can be elaborately designed on a single chip to realize multi-channel and even multi-target detection. Based on the abovementioned outstanding characteristics, many new techniques, such as colorimetry [19], electrochemistry [20], surface-enhanced Raman scattering [21], and fluorescence [22], have been integrated with μPADs for quantitative analysis. To achieve good performance detection on the μPADs biosensor, various nanomaterials with excellent properties and/or signal amplification strategies are introduced [23,24]. Among them, DNA nanomaterials have also drawn considerable research attention owing to their highly editable and natural biocompatibility. For instance, G-quadruplex (G4) derived from G-base-rich oligonucleotide sequences, as an intriguing nanostructure stabilized by Hoogsteen hydrogen bonds, possess dramatic fluorescence enhancement properties for fluorescent dye, which has been widely used in biosensors [25]. In particular, thioflavin T (4-(3,6-dimethyl-1,3-benzothiazol-3-ium-2-yl)-N,N-dimethylaniline, ThT) has gained extensive attention owing to its water solubility, low cost, and facile and selective light-up nature for G4 structure [26]. For example, Zhou and co-workers developed a G4/ThT system with a target-triggered conformational switch strategy and achieved a sensitive turn-on fluorescence assay for the detection of alkaline phosphatase, with a detection limit of 0.503 U/L [27]. Tan et al. designed a label-free molecular beacon with two regions using G4 structure as fluorogenic transducer to activate ThT for selective detection of DNA, RNA, and protein [28]. Zhang et al. reported a CRISPR/Cas12a-enabled amplification-free strategy via the integration of T7 exonuclease-mediated recycling amplification and split G4/ThT for RNA diagnostics [29]. Nevertheless, the fixation of structure-specific nucleic acid materials in anisotropic paper fibers was a challenging process, and they often became buried within the pores of the paper fibers, which consequently resulted in their ineffectiveness for sensing applications. In particular, well-defined self-assembled DNA tetrahedron (tetrahedral DNA framework, TDF) is a permission material to overcome this bottleneck, and it has been widely utilized for sensor construction [30]. This is due to its own excellent characteristics in terms of its splendid structural rigidity, excellent nanoscale controllability, and high productivity [31,32]. These outstanding properties prove advantageous for its meticulous regulation of the orientation and distribution of anchored probes [33], which dramatically improve the target recognition efficiency of the system and, in turn, elevate the system’s sensitivity. For instance, Yu et al. developed a TDF-based electrochemiluminescence strategy that significantly improved the hybridization efficiency and the entropy-driven strand displacement reaction, realizing highly sensitive detection of miRNA-133a with a low detection limit of 0.33 fM [34]. By employing TDF as a rigid scaffold to controllably anchor an aptamer via the regulatability of the density and orientation, Zhang and co-workers constructed a paper-based electrochemical aptasensor for sensitive and rapid detection of fumonisin B1 [35].

Taking inspiration from the fact that the G4/ThT system requires no modification and is easy to manipulate, a G-rich sequence [36,37] extending from the G4-TDF vertex was employed as specific recognition element and signal enhancement unit. By using the rigid TDF to fix the G-rich sequence and adjusting its orientation and distribution density, the target recognition efficiency can be effectively improved. In this study, a G4-TDF functionalized origami multipath μPAD based on the intermolecular G4 structure conversion was first designed for fluorescent detection of K^+^. In the presence of K^+^, it could trigger the conversion of a G-rich sequence from a parallel G-quadruplex structure into an antiparallel G-quadruplex structure [38], resulting in a significantly improved fluorescence enhancement effect toward the fluorescent dye thioflavin T (ThT) [39]. The proposed strategy exhibited good performance for quantitative analysis of K^+^, indicating the potential of our method as a promising tool for personalized healthcare and clinical diagnostics.

## 2. Materials and Methods

### 2.1. Reagents and Materials

All oligonucleotides (see Supplementary Table S2 for specific sequences) were synthesized and HPLC-purified by Sangon Biotechnology Co., Ltd. (Shanghai, China). Trizma hydrochloride (Tris-HCl) buffer solution (pH 7.0), chitosan, Tween-20, and bovine serum albumin (BSA) were obtained from Sigma-Aldrich (St. Louis, MO, USA). Glutaraldehyde and potassium chloride (KCl) were purchased from Sinopharm Chemical Reagent Co., Ltd. (Shanghai, China). Thioflavin T (ThT), TM buffer (pH 7.5), streptavidin, and phosphate buffer solution (1 × PBS) were provided by Sangon Biotechnology Co., Ltd. (Shanghai, China). PBST solution (0.05% Tween-20 PBS, pH 7.2) was prepared as washing buffer. Human serum was obtained from Beijing Lambolide Trading Co., Ltd. (Beijing, China). Whatman No. 1 filter paper (46 cm × 57 cm) was purchased from GE Healthcare Worldwide (Shanghai, China). All the solutions were prepared using ultra-pure deionized water (18.25 MΩ, UPD-II-10T, Chengdu, Sichuan, China).

### 2.2. Instrumentation

Fluorescence spectra were recorded on a Hitachi F-7000 fluorescence spectrophotometer (Tokyo, Japan). Circular dichroism (CD) spectra were obtained using a Chirascan circular dichroism spectrometer (Applied Photophysics Co., Ltd., Surrey, UK). The designed paper chips were printed using a wax printer (XEROX Phaser 8560DN), and then they were baked in an oven from Shanghai Jing Hong Laboratory Instrument Co., Ltd. (Shanghai, China).

### 2.3. Synthesis of G-Quadruplex/Tetrahedral DNA Framework (G4-TDF)

The G4-TDF was prepared according to a reported procedure, with minor modifications [40,41]. Briefly, a mixed solution of equimolar quantities of four strands in TM buffer was annealed at 95 °C for 7 min, followed by cooling to 4 °C for 25 min. The G4-TDF was characterized by agarose gel electrophoresis (Supplementary Figure S1).

### 2.4. Fabrication of the G4-TDF Functionalized Origami μPADs

The pattern of the origami μPADs was designed using Adobe Illustrator CS6 software and further printed with the aid of a wax-printing. Afterward, the wax-printed filter paper was baked in an oven (140 °C, 1 min) to generate the hydrophobic barrier. Then, the filter paper was cut into separate devices and was ready for modification of G4-TDF.

**Figure 1:**
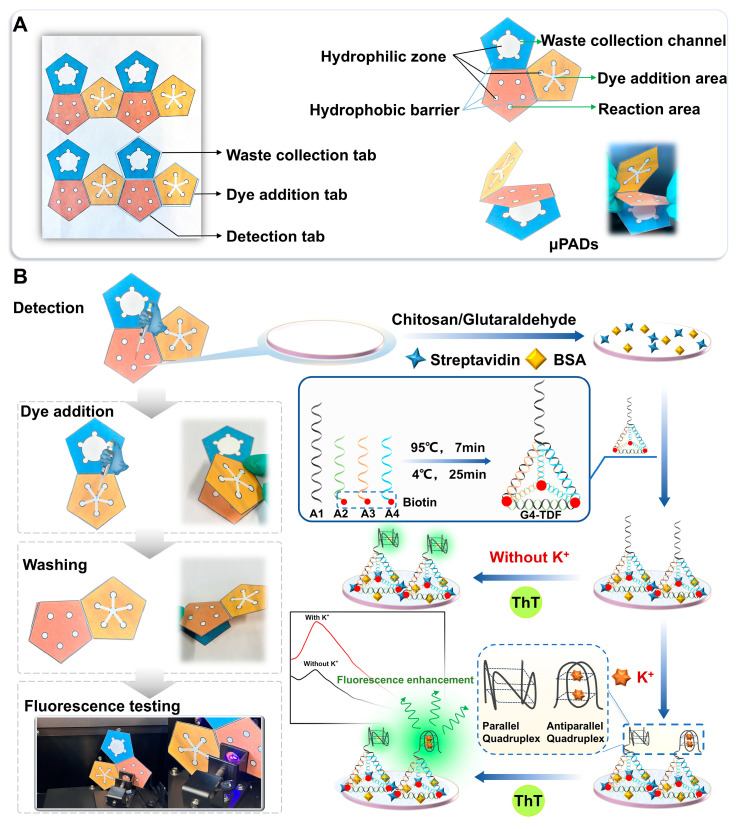
Schematic illustration of the designed origami μPADs (orange, blue, and yellow areas are the hydrophobic barriers) (**A**) and working principle of the G4-TDF functionalized μPADs for detection of K^+^ (**B**).

The device contained a detection tab (tab I), a dye addition tab (tab II), and a waste collection tab (tab III), as shown in Figure 1A. The detection tab consists of 5 reaction zones (5 mm in diameter, white in color). The dye addition tab is composed of six circular hydrophilic sites (5 mm in diameter, white in color) and five hydrophilic channels. When fluorescent dyes are added to the central site, they can reach the other five sites almost simultaneously along the hydrophilic channels (Supplementary Figure S2), and then they proceed to the reaction zones of the detection tab, thereby generating fluorescence reactions. The waste liquid collection layer consists of five circular sites (5 mm in diameter, white in color) and a circular waste liquid collection pool (25 mm in diameter, white in color), which can realize reaction and washing functions by folding the dye-addition layer, detection layer, and waste liquid collection layer up and down along the common edge of the three layers of paper chips.

Briefly, the prepared chitosan (2.5 × 10^−4^ g/mL) was added to tab I of the μPADs. After drying, glutaraldehyde (2.5%) was injected and reacted at room temperature for 2 h to form an aldehyde-rich chitosan film on the surface of the tab I. Rinsed the tab I with PBST to remove remaining unreacted reagents. Subsequently, 7 μL streptavidin (1 × 10^−4^ g/mL) was injected to react for 15 min, following by washing with PBST. After that, 7 μL BSA (0.5%) was added to block the non-specific sites on the tab I for 10 min and washed with PBST. Finally, 7 μL G4-TDF (5.0 × 10^−7^ M) was introduced and incubated for 20 min, and the designed G4-TDF functionalized μPADs were obtained after washing with PBST and stored for later use.

### 2.5. G4-TDF Functionalized Origami μPADs for Detection of K^+^

In total, 7 μL of KCl with different concentrations was dipped on tab I and incubated for 15 min, followed by washing thoroughly with PBST. Subsequently, tab II made contact with tab I via folding. Then, 7 μL (The estimated amount that arrived at each reaction zone) of ThT (1 μM) was added to tab II to act as a fluorescence signal indicator. After 10 min, the fluorescent spectra were collected with the utilization of the Hitachi F-7000 fluorescence spectrophotometer. The fluorescence spectrometer operated under an excitation wavelength at 425 nm.

### 2.6. The Real Sample Analysis

To demonstrate the practical performance of the developed origami μPADs for potassium analysis in real body fluids, we used them to detect K^+^ in human serum samples. Different concentrations of K^+^ standard solutions were added to human serum, and the labeled samples were analyzed according to the abovementioned detection methods. Human serum should be diluted fivefold when used.

## 3. Results and Discussion

### 3.1. Principle of Fluorescent Assay and Feasibility of the Origami μPADs for Detection of K^+^

Figure 1 shows the construction of G4-TDF functionalized μPADs and the principle of fluorescence strategy for the detection of K^+^. This μPADs was composed of three tabs: a detection tab (tab I), dye addition tab (tab II), and waste collection tab (tab III). In each layer, the white areas are the hydrophilic region, and the colored areas are the hydrophobic region. To effectively implement the fluorescent strategy, a versatile rigid structure tetrahedron with G-rich sequence was designed, aiming to realize target identification and fluorescence signal output. The rigid structure G4-TDF could accurately adjust the distance and orientation of recognition ligand G-rich sequence, which effectively avoided the sequence entanglement and greatly improved the identification efficiency of the system. Initially, the fluorescence dye, ThT, had the ability to bind to G-rich sequences within G4-TDF. This binding process led to the formation of a parallel G4 structure, consequently yielding a weak fluorescence signal output. Meanwhile, in the presence of K^+^, it could induce the G-rich sequence to form an antiparallel G4 structure [42]. In this case, the system is a mixture of parallel and antiparallel G4 structures. Subsequently, tab II was placed over tab I through folding manipulation (Figure 1B). After the fluorescence dye, ThT, was added to the center circular hydrophilic area on tab II, ThT could flow along the well-defined hydrophilic channels to the circular hydrophilic areas on the edge of tab II. Then through capillary action, it could further flow to the reaction zones on tab I, generating an enhanced fluorescent signal. This process enabled the fluorescent detection of K^+^.

**Figure 2:**
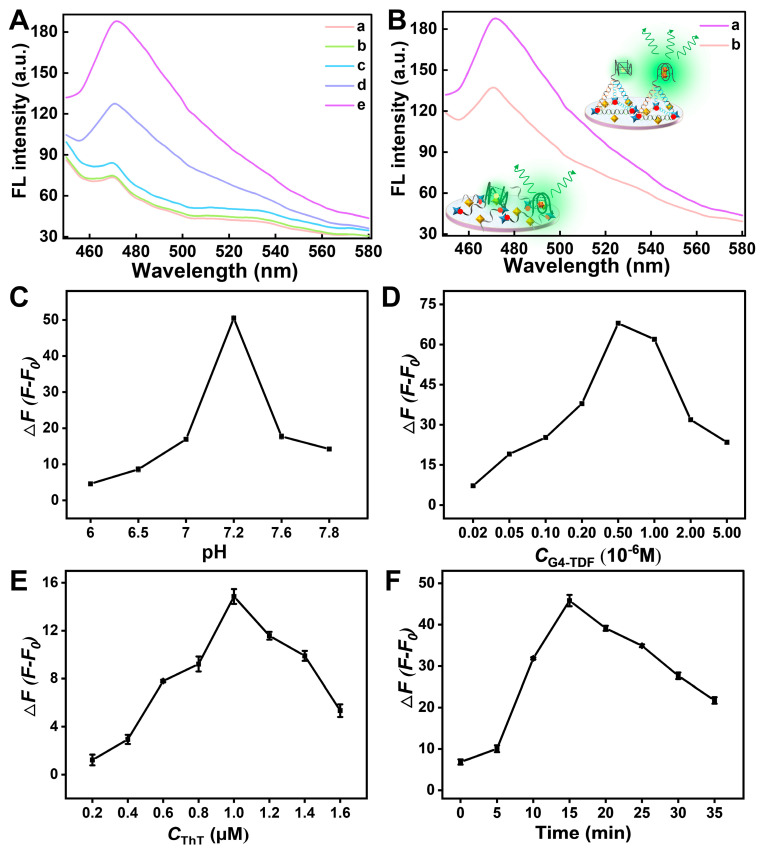
(**A**) Fluorescence spectra of the designed origami μPADs under different conditions: (**a**) μPADs; (**b**) μPADs + G4-TDF; (**c**) μPADs + G4-TDF + K^+^; (**d**) μPADs + G4-TDF + ThT; and (**e**) μPADs + G4-TDF + K^+^+ ThT. (**B**) Fluorescence spectra of the designed origami μPADs under different systems: (**a**) μPADs + G4-TDF + K^+^+ ThT, and (**b**) μPADs + C-DNA + K^+^+ ThT. (**C**–**F**) Optimization of experimental parameters. Effects of the pH of the buffer (**C**), the concentration of G4-TDF (**D**), the concentration of ThT (**E**), and reaction time (**F**) on K^+^ fluorescence sensing origami μPADs. (The concentration of K^+^ was 5 mM).

Primarily, the formation of G4-TDF was investigated by agarose gel electrophoresis. As expected, compared to single strand (lane 1 or lane 2) and the other combinations of two and three fragments (lanes 3 and 4), the assembly G4-TDF (lane 5) showed a distinct reduction in electrophoretic mobility, suggesting the successful assembly of G4-TDF compounds (Supplementary Figure S1). Additionally, the CD experiment was carried out to prove the formation of G4 structure. As can be seen from Supplementary Figure S3, the CD spectra had a positive peak at about 274 nm and a negative peak at about 248 nm, matching the characteristic of the formation of the G4 structure [42]. Then, to verify the supposition, the fluorescence curves of different conditions were investigated. As shown in Figure 2A, the system reflects a very low signal for μPADs (a), μPADs + G4-TDF (b), and μPADs + G4-TDF + K^+^ (c). When ThT was added, a fluorescence light-up was observed owing to the fact that the cationic dye, ThT, could induce quadruplex folding in the extended single-stranded G-rich sequence of G4-TDF to form a parallel G-quadruplex structure. Strikingly, in the presence of K^+^/ThT (e), the remarkable fluorescence enhancement displayed owing to the cooperative stabilization of the antiparallel G-quadruplex structure induced by both K^+^ and ThT. Meanwhile, as illustrated in Figure 2B, the system with TDF as a support frame performs better in sensing than the single-stranded DNA. All of these results revealed that the ThT/G4-TDF system was in agreement with our expectations and could competently achieve the G-quadruplex structure conversion mechanism for selective detection of K^+^.

### 3.2. Optimization of Experimental Conditions

To achieve the desirable performance for the proposed μPADs, some vital parameters, including the pH value of the buffer, concentration of G4-TDF, concentration of ThT, and incubation time of K^+^, were fully optimized. Firstly, the pH value of the buffer solution was studied. As shown in Figure 2C, with the increase in the pH value, the fluorescence intensity changes, Δ*F* (Δ*F* = *F* − *F*_0_, where *F* and *F*_0_ represent the changed fluorescence intensity values in the presence and absence of K^+^, respectively), gradually increased with the increasing pH value and reached an inflection point at pH 7.2. Thus, pH 7.2 was adopted in the following experiment. Next, the concentration of G4-TDF was optimized. The G-rich sequence extending from G4-TDF could be induced to form parallel or antiparallel G-quadruplex structure by binding with ThT or ThT/K^+^ to produce fluorescence signal. Therefore, the amount of G4-TDF could directly affect the sensitivity of the system. From Figure 2D, we observed that the fluorescence intensity changes reached the maximum at a G4-TDF concentration of 5 × 10^−7^ M. Therefore, 5 × 10^−7^ M was selected as the optimal concentration of G4-TDF. Subsequently, the concentration of ThT was optimized. Figure 2E indicates that the Δ*F* value increased with increasing the concentration of ThT in the range of 0.2–1.0 μM, and decreased gradually with further increasing concentration. So, 1.0 μM was selected as the optimal condition. Finally, the reaction time between K^+^ and the G4-TDF probe was optimized. As illustrated in Figure 2F, the Δ*F* value undergoes a gradual increase within the range from 0 to 15 min. And the Δ*F* reaches its maximum value at 15 min, after which it begins to decline. Consequently, 15 min was identified as the optimal result for the K^+^ reaction time.

### 3.3. Performance Study of the Origami μPADs for Detection of K^+^

**Figure 3:**
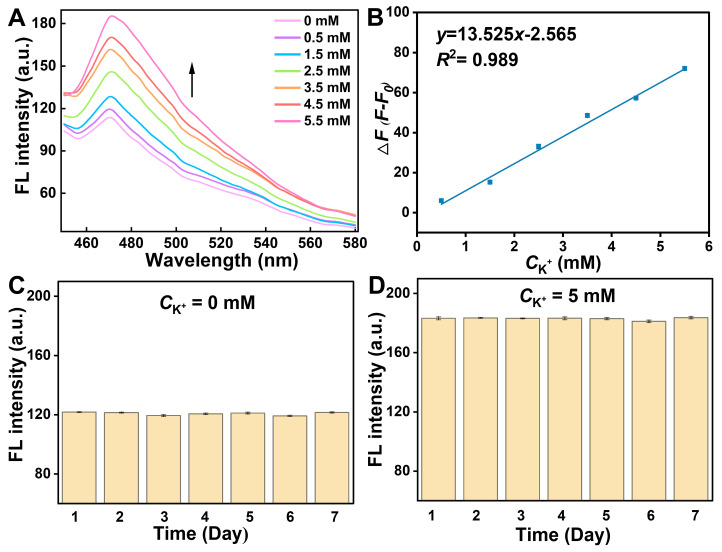
(**A**) Fluorescence spectra of the G4-TDF functionalized μPADs incubating with different concentrations of K^+^ (0–5.5 mM). (**B**) Linear relationship between Δ*F* and K^+^ concentrations. (**C,D**) Stability of the G4-TDF functionalized μPADs. (The concentrations of K^+^ were 0 mM (**C**) and 5 mM (**D**)).

**Figure 4:**
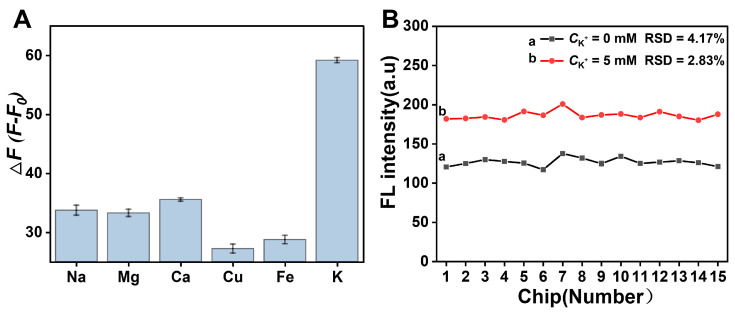
(**A**) Specificity of the constructed G4-TDF functionalized μPADs toward other potentially interfering ions (Na^+^, Mg^2+^, Ca^2+^, Cu^2+^, and Fe^3+^). The concentrations of the various metal ions were 5 mM. (**B**) Reproducibility of the G4-TDF functionalized μPADs.

The performance of the G4-TDF functionalized μPADs based on intermolecular G-quadruplex structure conversion was explored by measuring the fluorescence response to different concentrations of K^+^ under optimal conditions. As shown in Figure 3A, the fluorescence intensity rose with the increase in the K^+^ concentration from 0 to 5.5 mM, as more K^+^ was bound with G-rich sequence, and more of the parallel G-quadruplex structure was transformed into the antiparallel G-quadruplex structure. This antiparallel G-quadruplex structure is more stable and more conducive to enhancing the fluorescence of ThT. Figure 3B indicates that the Δ*F* was proportional to the concentration of K^+^ over the range of 0.5–5.5 mM. The limit of detection was estimated to be 0.2 mM (y_LOD_ = 3σ/k), which compared well with those previously reported method for detection of K^+^ (Table 1). This high sensitivity is attributed to the increased stability and fluorescence enhancement properties of antiparallel G-quadruplex structure toward ThT induced by K^+^. Furthermore, the stability of this strategy was evaluated through the intermittent fluorescence response tests. As illustrated in Figure 3C,D, the fluorescence signal of the G4-TDF functionalized μPADs remained stable over the course of seven days, demonstrating the excellent stability of our μPADs. Simultaneously, the specificity of the method toward K^+^ against other cations, including Na^+^, Mg^2+^, Ca^2+^, Cu^2+^, and Fe^3+^, was investigated. As shown in Figure 4A, an obvious fluorescence enhancement was observed only when K^+^ was present, while other cations showed no significant fluorescence intensity increase, indicating the excellent specificity of the method for detecting K^+^. Then, the reproducibility of the designed μPADs was explored at different K^+^ concentrations, including 0 mM and 5 mM. The Figure 4B showed that the relative standard deviation (RSD) from 15 different paper chips was less than 5% for each concentration of K^+^. Moreover, Supplementary Figure S4 demonstrated that the RSD of the Δ*F* values from the 15 groups of paper chips was less than 10%. These results indicated that the designed G4-TDF functionalized μPAD has satisfactory reproducibility.

### 3.4. Detection of K^+^ in Actual Samples

In order to assess the application potential of the G4-TDF functionalized μPADs, a recovery experiment was carried out by spiking various concentrations of K^+^ into human serum. As can be seen from Supplementary Table S1, the calculated recoveries were from 90.4% to 107.2%, and the RSD values do not exceed 2.05%. These results demonstrated that the designed G4-TDF functionalized μPADs is a simple and reliable analytical tool with broad potential for the rapid detection of K^+^ content in clinical analysis.

## 4. Conclusions

In summary, an efficient G4-TDF functionalized origami μPAD was designed for sensitive detection of K^+^ based on the target-induced intermolecular G-quadruplex structure conversion through the strong specific binding between G-quadruplex and K^+^/ThT. We integrated the G4/ThT system with the rigid structure of TDF to fabricate a dual-function component serving as both a specific recognition element and a signal enhancement unit, which was then coupled with the origami μPADs platform for detection of K⁺.

More importantly, the proposed μPADs platform exhibited a low detection limit of 0.2 mM, excellent specificity, desirable reproducibility, and good stability. Therefore, we believe that this simple, practical, low-cost, portable, and convenient G4-TDF functionalized origami μPAD had a great potential to be adopted as a significant alternative protocol in personalized healthcare and clinical diagnostics.

## Figures and Tables

**Table 1:** Comparison with previously reported works for K^+^ determination.

Method	System	Linear Range	LOD	Reference
Optical	Using photoluminescent single-walled carbon nanotubes (SWCNTs) encapsulated in polymers that contain potassium chelating moieties	5.0–7.0 mM	0.39 mM	[43]
Electrochemistry	Based on antimony tin oxide (ATO)–Prussian blue (PB) screen-printed electrode (SPE) and PEDOT-PB modified glassy carbon electrode	0.1–10 mM	1.1 mM	[44]
Near-infrared ray (NIR)	Based on remote-controlled “lock−unlock” nanosystem using dual-stranded aptamer precursor as recognition molecules	0–60 mM	4 mM	[45]
Fluorescence	A ratiometric fluorescent microsensor based on IPG4–silica microparticles	0–30 mM	9.51 mM	[46]
Fluorescence	Based on aptamer and pyrene-labeled fluorescent probes	0.6–20 mM	0.4 mM	[47]
Fluorescence	G4-TDF functionalized μPADs based on intermolecular structure conversion	0.5–5.5 mM	0.2 mM	This work

## Data Availability

All the data are contained in the article and in the Supplementary Materials.

## Supplementary Materials

The following supporting information can be downloaded at https://www.mdpi.com/article/10.3390/bios15040223/s1, Figure S1: Agarose gel electrophoretic characterization of G4-TDF. Lane 1, A1; lane 2, A2; lane 3, A1+A2; lane 4, A1+A2+A3; and lane 5, G4-TDF (A1+A2+A3+A4); Figure S2: (A) The appearance of the dye addition tab (tab II) under daylight when the fluorescence dye ThT was added. Upon the introduction of the fluorescent dye thioflavin T (ThT) at the central site of tab II, the ThT-containing solution underwent a rapid propagation along the well-defined hydrophilic channels, driven by capillary forces. (B) The images of the five reaction zones within detection the tab under UV light at different times after the fluorescence dye ThT was added; Figure S3: Circular dichroism spectra of G4-TDF/ThT. The positive peak at about 274 nm and the negative peak at about 248 nm indicating the formation of G4 structure; Figure S4: Reproducibility of the proposed fluorescence μPADs sensing platform (Δ*F* = *F* − *F*_0_, where *F* and *F*_0_ represent the fluorescence intensity changed values in the presence and absence of K^+^); Table S1: Recovery results of this assay for K^+^ detection in human serum sample; Table S2: Sequences of oligonucleotides used in this work.

## References

[1] Sun H.B., Weaver C.M. (2020). Rise in potassium deficiency in the US population linked to agriculture practices and dietary potassium deficits. J. Agric. Food Chem..

[2] Debnath M., Chakraborty S., Kumar Y.P., Chaudhuri R., Jana B., Dash J. (2020). Ionophore constructed from non-covalent assembly of a G-quadruplex and liponucleoside transports K^+^-ion across biological membranes. Nat. Commun..

[3] Wang G.F., Chen L., Zhu Y.H., He X.P., Xu G., Zhang X.J. (2014). Development of an electrochemical sensor based on the catalysis of ferrocene actuated hemin/G-quadruplex enzyme for the detection of potassium ions. Biosens. Bioelectron..

[4] Cheng C.J., Kuo E., Huang C.L. (2013). Extracellular potassium homeostasis: Insights from hypokalemic periodic paralysis. Semin. Nephrol..

[5] Wang Z.Q., Pan T.T., Shen M., Liao J.X., Tian Y.Q. (2023). Cross-conjugated polymers as fluorescent probes for intracellular potassium ion detection. Sens. Actuators B.

[6] Chen Z.B., Guo J.X., Ma H., Zhou T., Li X.X. (2014). A simple colorimetric sensor for potassium ion based on DNA G-quadruplex conformation and salt-induced gold nanoparticles aggregation. Anal. Methods.

[7] Yan Y., Han B.Q., Zeng J., Zhou W.Y., Zhang T.J., Zhang J.T., Chen W.X., Zhang C.B. (2017). A candidate reference method for serum potassium measurement by inductively coupled plasma mass spectrometry. Clin. Chem. Lab. Med..

[8] Arnquist I.J., Hoppe E.W. (2017). The quick and ultrasensitive determination of K in NaI using inductively coupled plasma mass Spectrometry. Nucl. Instrum. Methods Phys. Res. Sect. A.

[9] García-Alegría A.M., Cáñez-Carrasco M.G., Serna-Félix M., Encinas Soto K.K., Gómez-Álvarez A. (2018). Estimation of uncertainty in the determination of serum electrolytes (Na, K, Ca, Mg) by flame atomic absorption spectroscopy. MAPAN-J. Metrol. Soc. I..

[10] Qu Z.C., Steinvall E., Ghorbani R., Schmidt F.M. (2016). Tunable diode laser atomic absorption spectroscopy for detection of potassium under optically thick conditions. Anal. Chem..

[11] Messele H.M., Asres Y.H., Hiruy B.Z. (2024). Determination of chemical elements of barley and teff using flame atomic absorption spectroscopy (FAAS). Appl. Radiat. Isot..

[12] Gamela R.R., Barrera E.G., Duarte Á.T., Boschetti W., da Silva M.M., Vale M.G.R., Dessuy M.B. (2019). Fast sequential determination of Zn, Fe, Mg, Ca, Na, and K in infant formulas by high-resolution continuum source flame atomic absorption spectrometry using ultrasound-assisted extraction. Food Anal. Methods.

[13] Eum N.S., Lee S.H., Lee D.R., Kwon D.K., Shin J.K., Kim J.H., Kang S.W. (2003). K^+^-ion sensing using surface plasmon resonance by NIR light source. Sens. Actuators B.

[14] Alfonso A., Pazos M.J., Fernández-Araujo A., Tobio A., Alfonso C., Vieytes M.R., Botana L.M. (2014). Surface plasmon resonance biosensor method for palytoxin detection based on Na^+^,K^+^-ATPase affinity. Toxins.

[15] Kanyanee T., Tianrungarun K., Somboot W., Puangpila C., Jakmunee J. (2022). Open tubular capillary ion chromatography with online dilution for small ions determination in drinks. Food Chem..

[16] Michalski R., Lyko A. (2016). Research onto the contents of selected inorganic ions in the dialysis fluids and dialysates by using ion chromatography. J. Liq. Chromatogr. Relat. Technol..

[17] Li K., Wang J., Wang J.Q., Zheng Z., Liu X.P., Wang J.K., Zhang C.J., He S.S., Wei H., Yu C.Y. (2024). A programmable microfluidic paper-based analytical device for simultaneous colorimetric and photothermal visual sensing of multiple enzyme activities. Anal. Chem..

[18] Moulahoum H. (2023). Dual chromatic laser-printed microfluidic paper-based analytical device (μPAD) for the detection of atrazine in water. ACS Omega.

[19] Xiong X.L., Zhang J.L., Wang Z., Liu C.C., Xiao W.D., Han J.F., Shi Q.F. (2020). Simultaneous multiplexed detection of protein and metal ions by a colorimetric microfluidic paper-based analytical device. BioChip J..

[20] Ming T., Cheng Y., Xing Y., Luo J.P., Mao G., Liu J.T., Sun S., Kong F.L., Jin H.Y., Cai X.X. (2021). Electrochemical microfluidic paper-based aptasensor platform based on a biotin–streptavidin system for label-free detection of biomarkers. ACS Appl. Mater. Interfaces.

[21] Zhuang J.W., Zhao Z.Y., Lian K., Yin L.J., Wang J.J., Man S.L., Liu G.Z., Ma L. (2022). SERS-based CRISPR/Cas assay on microfluidic paper analytical devices for supersensitive detection of pathogenic bacteria in foods. Biosens. Bioelectron..

[22] Jiang H.E., Zhang Q., Li N.H., Li Z.J., Chen L.J., Yang F.Q., Zhao S.Q., Liu X.H. (2024). All-in-one strategy for the nano-engineering of paper-based bifunctional fluorescent platform for robustly-integrated real-time monitoring of food and drinking-water safety. J. Hazard. Mater..

[23] Zhou C.X., Cui K., Liu Y., Hao S.J., Zhang L.N., Ge S.G., Yu J.H. (2021). Ultrasensitive microfluidic paper-based electrochemical/visual analytical device via signal amplification of Pd@hollow Zn/Co core–shell ZIF67/ZIF8 nanoparticles for prostate-specific antigen detection. Anal. Chem..

[24] Liu L.Y., Yang D.T., Liu G.Z. (2019). Signal amplification strategies for paper-based analytical devices. Biosens. Bioelectron..

[25] Ma L., Han X., Xia L., Qu F.L., Kong R.M. (2020). A label-free G-quadruplex-based fluorescence assay for sensitive detection of alkaline phosphatase with the assistance of Cu^2+^. Spectrochim. Acta A.

[26] Khusbu F.Y., Zhou X., Chen H.C., Ma C.B., Wang K.M. (2018). Thioflavin T as a fluorescence probe for biosensing applications. Trends Anal. Chem..

[27] Zhou X., Khusbu F.Y., Chen H.C., Ma C.B. (2020). A turn-on fluorescence assay of alkaline phosphatase activity based on an enzyme-triggered conformational switch of G-quadruplex. Talanta.

[28] Tan X.H., Wang Y., Armitage B.A., Bruchez M.P. (2014). Label-free molecular beacons for biomolecular detection. Anal. Chem..

[29] Zhang D.C., Tian B.S., Ling Y., Ye L., Xiao M., Yuan K.X., Zhang X.Q., Zheng G.S., Li X.Y., Zheng J.D. (2024). CRISPR/Cas12a-powered amplification-free RNA diagnostics by integrating T7 exonuclease-assisted target recycling and split G-quadruplex catalytic signal output. Anal. Chem..

[30] Zou G.Y., Bi F., Yu Y.L., Liu M.X., Chen S. (2024). Tetrahedral DNA-based ternary recognition ratiometric fluorescent probes for real-time in situ resolving lysosome subpopulations in living cells via Cl^−^, Ca^2+^, and pH. Anal. Chem..

[31] Kansara K., Singh R., Yadav P., Mansuri A., Kumar A., Bhatia D. (2023). Lipid modification of DNA tetrahedrons enhances cellular uptake, migration, and in vivo uptake. ACS Appl. Nano Mater..

[32] Wang Q., Ma Y.X., Lu Z.W., Yu H.Y., Li Z. (2022). Co-delivery of chemotherapeutic drugs and immune adjuvants by nanoscale DNA tetrahedrons for synergistic cancer therapy. ACS Appl. Nano Mater..

[33] Chen X., Huang J., Zhang S., Mo F., Su S.S., Li Y., Fang L.C., Deng J., Huang H., Luo Z.X. (2019). Electrochemical biosensor for DNA methylation detection through hybridization chain-amplified reaction coupled with a tetrahedral DNA nanostructure. ACS Appl. Mater. Interfaces.

[34] Yu L.Y., Zhu L.P., Yan M.X., Feng S.N., Huang J.S., Yang X.R. (2021). Electrochemiluminescence biosensor based on entropy-driven amplification and a tetrahedral DNA nanostructure for miRNA-133a detection. Anal. Chem..

[35] Zhang X.B., Li Z.R., Hong L., Wang X.W., Cao J.J. (2023). Tetrahedral DNA nanostructure-engineered paper-based electrochemical aptasensor for fumonisin B1 detection coupled with Au@Pt nanocrystals as an amplification label. J. Agric. Food Chem..

[36] Pal S., Naik A., Rao A.J.A., Chakraborty B., Varma M.M. (2022). Aptamer-DNA origami-functionalized solid-state nanopores for single-molecule sensing of G-quadruplex formation. ACS Appl. Nano Mater..

[37] Chitbankluai K., Thavarungkul P., Kanatharana P., Kaewpet M., Buranachai C. (2022). Newly found K^+^-thioflavin T competitive binding to DNA G-quadruplexes and the development of a label-free fluorescent biosensor with extra low detection limit for K^+^ determination in urine samples. Spectrochim. Acta A.

[38] Yang L., Qing Z.H., Liu C.H., Tang Q., Li J.S., Yang S., Zheng J., Yang R.H., Tan W.H. (2016). Direct fluorescent detection of blood potassium by ion-selective formation of intermolecular G-quadruplex and ligand binding. Anal. Chem..

[39] Liu H.S., Zhang X., Li X.R., Wu H.S., Shi Y.W., Lu W. (2024). A G-quadruplex/thioflavin T-based label-free biosensor to detect ClO^−^ in stress-induced hypertension. Spectrochim. Acta A.

[40] Hong S.B., Jiang W.D., Ding Q.F., Lin K.L., Zhao C.C., Wang X.D. (2023). The current progress of tetrahedral DNA nanostructure for antibacterial application and bone tissue regeneration. Int. J. Nanomed..

[41] Ren W.J., Pang J.R., Ma R.R., Liang X.J., Wei M., Suo Z.G., He B.S., Liu Y. (2022). A signal on-off fluorescence sensor based on the self-assembly DNA tetrahedron for simultaneous detection of ochratoxin A and aflatoxin B1. Anal. Chim. Acta.

[42] Mohanty J., Barooah N., Dhamodharan V., Harikrishna S., Pradeepkumar P.I., Bhasikuttan A.C. (2013). Thioflavin T as an efficient inducer and selective fluorescent sensor for the human telomeric G-quadruplex DNA. J. Am. Chem. Soc..

[43] Dewey H.M., Mahmood N., Abello S.M., Sultana N., Jones J., Gluck J.M., Budhathoki-Uprety J. (2024). Development of optical nanosensors for detection of potassium ions and assessment of their biocompatibility with corneal epithelial cells. ACS Omega.

[44] Leau S.A., Lete C., Marin M., del Campo F.J., Diaconu I., Lupu S. (2023). Electrochemical sensors based on antimony tin oxide-prussian blue screen-printed electrode and PEDOT-prussian blue for potassium ion detection. J. Solid State Electrochem..

[45] Cui M.R., Chen L.X., Li X.L., Xu J.J., Chen H.Y. (2020). NIR remote-controlled “lock–unlock” nanosystem for imaging potassium ions in living cells. Anal. Chem..

[46] Colella F., Forciniti S., Onesto V., Grasso G., Iuele H., Gigli G., del Mercato L.L. (2024). A fluorescent ratiometric potassium sensor based on IPG4-silica microparticles for selective detection and fluorescence imaging of potassium cations. J. Mater. Chem. B.

[47] Shi C., Gu H.X., Ma C.P. (2010). An aptamer-based fluorescent biosensor for potassium ion detection using a pyrene-labeled molecular beacon. Anal. Biochem..

