# Supplementary material for: Intermolecular Structure Conversion-Based G4-TDF Nanostructures Functionalized μPADs for Fluorescent Determination of Potassium Ion in Serum
Source: Biosensors (Basel). 2025 Mar 31;15(4):223. doi: 10.3390/bios15040223 (PMC12024665; doi:10.3390/bios15040223)
Supplement: Supplementary file 1 [file biosensors-15-00223-s001.zip › biosensors-3503364-supplementary.pdf]

Supplementary Materials

Intermolecular Structure Conversion-Based G4-TDF Nanostructures Functionalized μPADs for Fluorescent Determination of Potassium Ion in Serum

Mengqi Wang [1], Xiuli Fu [1], Yixuan Liu [1], Zhiyang Zhang [2], Chenyu Jiang [3,*] and Dean Song [4,*]

[1] School of Chemistry and Chemical Engineering, Yantai University, Yantai 264005, China; 202200369065@s.ytu.edu.cn (M.W.); fuxiuli@ytu.edu.cn (X.F.); liuyx2025@foxmail.com (Y.L.)
[2] Coastal Zone Ecological Environment Monitoring Technology and Equipment Shan-dong Engineering Research Center, Shandong Key Laboratory of Coastal Environ-mental Processes, CAS Key Laboratory of Coastal Environmental Processes and Ecological Remediation, Yantai Institute of Coastal Zone Research, Chinese Academy of Sciences, Yantai 264003, China; zyzhang@yic.ac.cn
[3] Suzhou Institute of Biomedical Engineering and Technology, Chinese Academy of Sciences, Suzhou 215163, China
[4] Tobacco Research Institute, Chinese Academy of Agricultural Sciences, Qingdao 266101, China
* Correspondence: jiangcy@sibet.ac.cn (C.J.); songdean@caas.cn (D.S.)

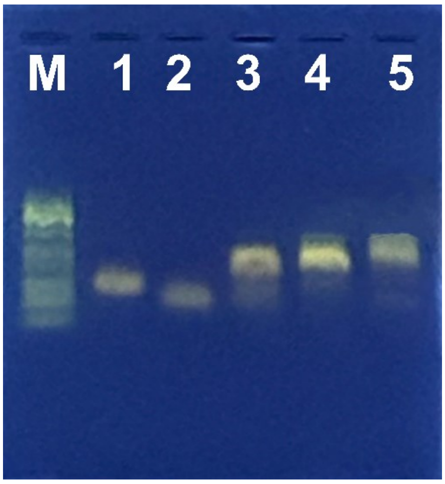

Figure S1. Agarose gel electrophoretic characterization of G4-TDF. M: Marker, Lane 1: A1, lane 2: A2, lane 3: A1+A2, lane 4: A1+A2+A3, lane 5: G4-TDF (A1+A2+A3+A4).

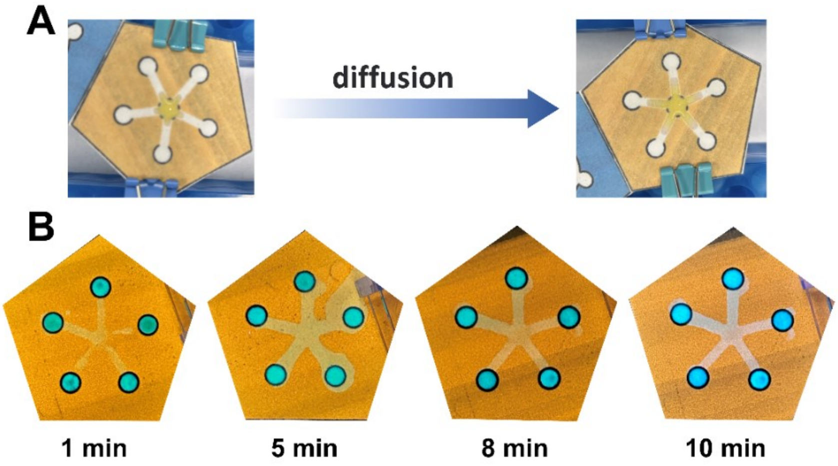

Figure S2. (A) The appearance of the dye addition tab (tab Ⅱ) under daylight when the fluorescence dye ThT

was added. Upon the introduction of the fluorescent dye thioflavin T (ThT) at the central site of tab Ⅱ, the ThT-containing solution underwent a rapid propagation along the well-defined hydrophilic channels, driven by capillary forces. (B) The images of the five reaction zones within detection tab under UV light at different times after the fluorescence dye ThT was added.

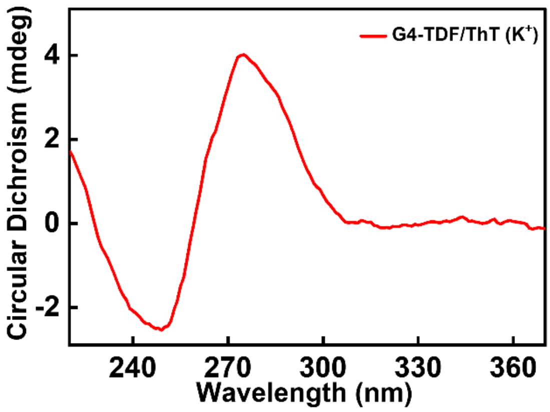

Figure S3. Circular dichroism spectra of G4-TDF/ThT. The positive peak at about 274 nm and the negative peak at about 248 nm indicating the formation of G4 structure.

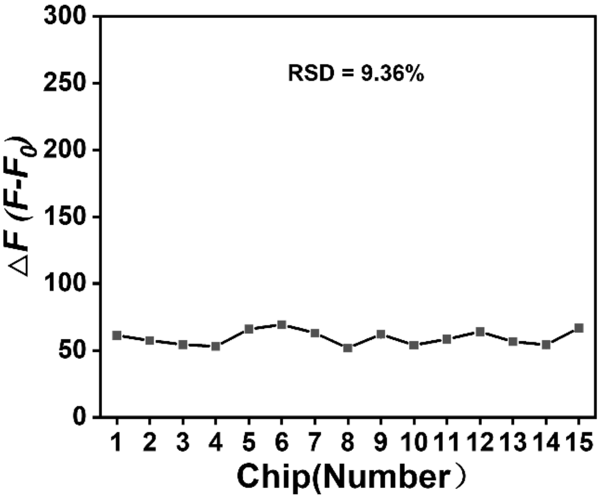

Figure S4. Reproducibility of the proposed fluorescence μPADs sensing platform ($\Delta F = F - F_0$, where F and F_0 represent the fluorescence intensity changed values in the presence and absence of K^+).

Table S1. Recovery results of this assay for K^+ detection in human serum sample

Sample	Added (mM)	Found (mM)	Recovery ± RSD (%)
	2.50	2.68	107.2%±1.79
Serum	3.50	3.53	100.9%±2.05
	4.50	4.07	90.4%±0.45

Table S2. Sequences of oligonucleotides used in this work

Type	DNA sequences (5′-3′)
A1	ACATTCCTAAGTCTGAAACATTACAGCTTGCTACACGAGA AGAGCCGCCATAGTATTTTTTTTTTGGGTTAGGGTTAGGGTTAGGG
A2	Biotin-TATCACCAGGCAGTTGACAGTGTAGCAAGCTGTA ATAGATGCGAGGGTCCAATAC
A3	Biotin-TCAACTGCCTGGTGATAAAACGACACTACGTGGG AATCTACTATGGCGGCTCTTC
A4	Biotin-TTCAGACTTAGGAATGTGCTTCCCACGTAGTGTC GTTTGTATTGGACCCTCGCAT
C-DNA	Biotin-TTTTTTTTTTTTTTTTTTTTGGGTTAGGGTTAGGG TTAGGG
